# Screening Peptide-Binding Partners for GenX via Phage Display

**DOI:** 10.3390/ijms25052686

**Published:** 2024-02-26

**Authors:** Kameron Burton, Samaneh Ghadami, Kristen Dellinger, Bo Wang, Ming Dong

**Affiliations:** 1 Department of Chemistry, North Carolina Agricultural and Technical State University, Greensboro, NC 27411, USA; 2 Department of Nanoengineering, Joint School of Nanoscience and Nanoengineering, North Carolina Agricultural and Technical State University, Greensboro, NC 27411, USA; 3 Department of Chemistry and Chemical Engineering, Florida Institute of Technology, Melbourne, FL 32901, USA; 4 Department of Chemistry and Biochemistry, University of North Carolina Wilmington, Wilmington, NC 28403, USA

**Keywords:** PFAS, phage display, GenX, MD simulation, NMR

## Abstract

Per- and poly-fluoroalkyl substances (PFAS), such as GenX, are a class of highly stable synthetic compounds that have recently become the focus of environmental remediation endeavors due to their toxicity. While considerable strides have been made in PFAS remediation, the diversity of these compounds, and the costs associated with approaches such as ion exchange resins and advanced oxidation technologies, remain challenging for widespread application. In addition, little is known about the potential binding and impacts of GenX on human proteins. To address these issues, we applied phage display and screened short peptides that bind specifically to GenX, with the ultimate goal of identifying human proteins that bind with GenX. In this study we identified the amino acids that contribute to the binding and measured the binding affinities of the two discovered peptides with NMR. A human protein, ankyrin-repeat-domain-containing protein 36B, with matching sequences of one of the peptides, was identified, and the binding positions were predicted by docking and molecular dynamics simulation. This study created a platform to screen peptides that bind with toxic chemical compounds, which ultimately helped us identify biologically relevant molecules that could be inhibited by the GenX, and also provided information that will contribute to future bioengineered GenX-binding device design.

## 1. Introduction

Per- and polyfluoroalkyl substances (PFAS) are synthetic chemicals with extremely stable carbon-fluorine bonds, which makes them very persistent in the environment. Further, PFAS has been shown to exhibit detrimental impacts on human health, showing accumulation in the blood and liver and causing adverse human health effects [1,2,3,4,5,6,7,8,9,10,11].

**Figure 1:**
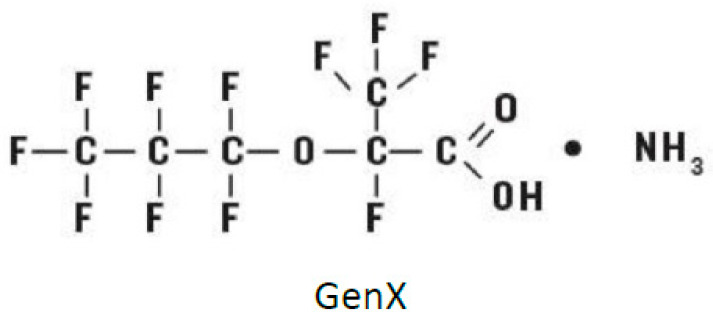
Structure of GenX.

GenX (Figure 1) has a smaller molecular weight compared to others in the PFAS family. Recent studies have shown the ubiquitous appearance of GenX in our environment, e.g., groundwater and drinking water, and animal studies have highlighted potentially negative effects, including risks for the kidney and immune system, as well as for the liver and pancreas [12,13]. Moreover, GenX has been shown to impact several disease-related human proteins, e.g., multidrug resistance-associated protein 2, at the blood-brain barrier, the transport activity of the human breast cancer resistance protein (BCRP), and P-glycoprotein (P-gp) [1,2,3,4,5]. Despite increased commercial production of GenX, there is limited knowledge of its role in the human body at the molecular level, and more exploration is needed to develop recycling solutions.

Phage display can be applied to discover de novo binding partners for small molecules [14], and short peptides have been screened to target active bioengineered materials with high specificity [15]. In this paper, we screened short peptides (7mer) that recognize GenX, using in vitro phage display to identify amino-acid sequences bound to GenX and analyze the binding affinity with NMR. The sequences obtained from the phage display were then used to blast protein sequences to identify human protein that binds with GenX. Molecular dynamic (MD) simulations were performed to analyze the binding mode of GenX with the identified protein.

## 2. Results and Discussion

**Figure 2:**
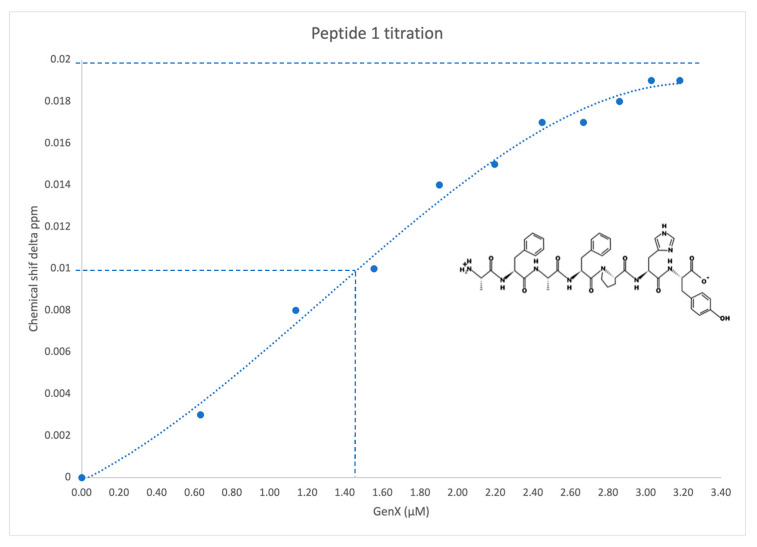
Titration of peptide 1 (AFAFPHY) against GenX, as followed by 1H NMR spectroscopy, revealed a Kd of ~1.5 µM. The 50% completed chemical shift was labelled with dash line which gave the estimated Kd.

**Figure 3:**
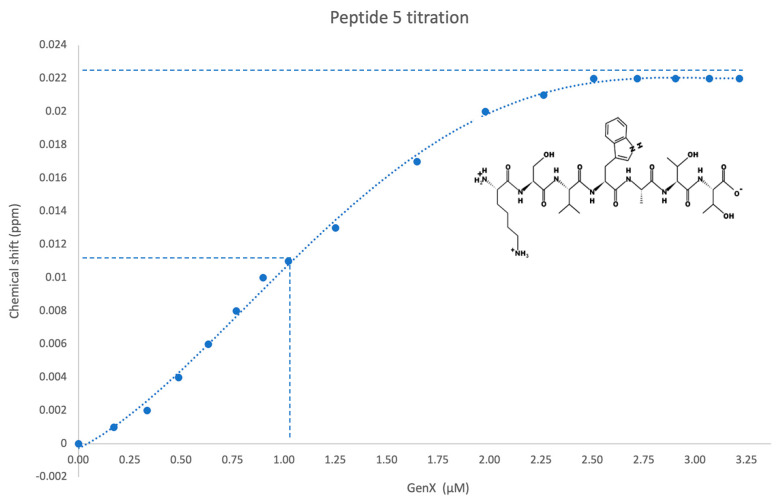
Titration of peptide 5 (KSVWATT) against GenX as followed by 1H NMR spectroscopy revealed a Kd of 1 µM. The 50% completed chemical shift was labelled with dash line which gave the estimated Kd.

**Figure 4:**
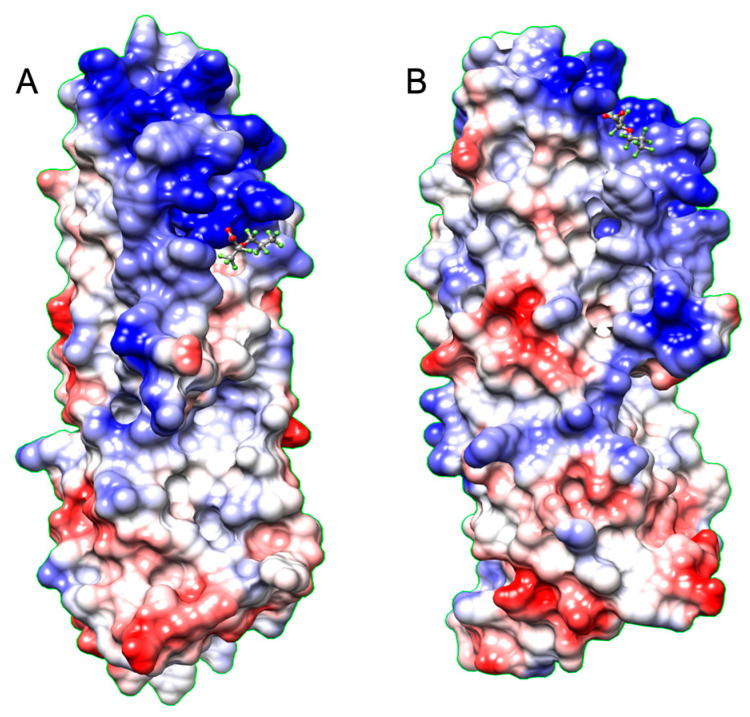
Overview of the two binding sites, site 1 (**A**) and site 2 (**B**), of GenX on protein 36B from MD simulation. The blue, red and white colors represent positive, negative, and neutral charged regions, respectively.

**Figure 5:**
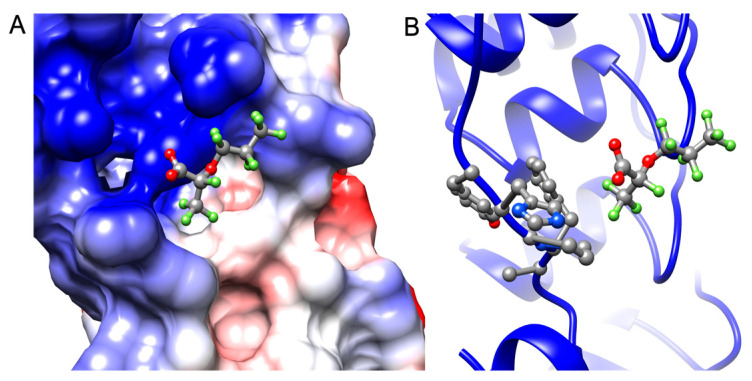
(**A**) The close-up of the GenX binding site 1 on protein 36B displayed on the surface. (**B**) The close-up of how GenX binds to protein 36B at site 1, which contains the FAFPHY sequence—this is shown in ball and stic, where the His13 is in close contact with the carboxyl group of GenX. The blue, red and white colors represent positive, negative, and neutral charged regions, respectively.

**Figure 6:**
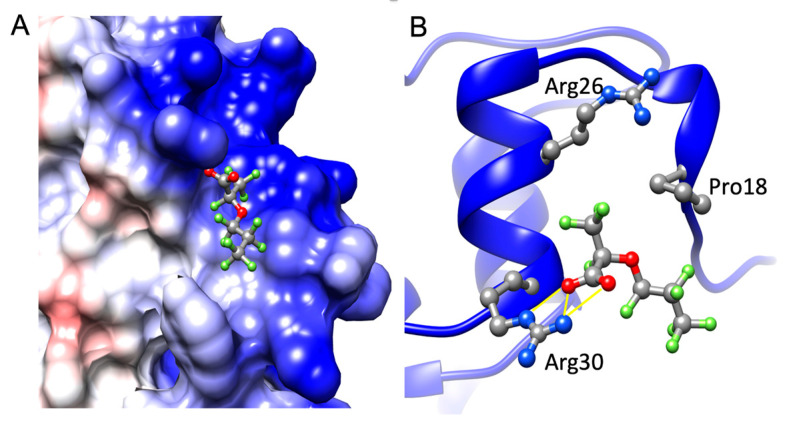
(**A**) The close-up of the GenX binding site 2 on 36B displayed on the surface. (**B**) The close-up of how GenX binds to the 36B at site 2. Pro18, Arg26 and Arg30 are in close contact with GenX. There are hydrogen bonds between Arg30 and the GenX carboxylate group. The blue, red and white colors represent positive, negative, and neutral charged regions, respectively.

The phage display experiment identified four de novo peptides that bind to GenX (Table 1). The NMR titration experiment showed peptide 1 and peptide 5 bound with GenX, with an affinity of 1.6 µM and 1 µM, respectively (Table 1, Figure 2 and Figure 3). After sequence blasting there was a protein sequence match for peptide #1, the N-term (FAFPHY) of a human protein—the ankyrin-repeat-domain-containing protein 36B, which functions to mediate protein-protein interactions, and is directly involved in the development of human cancer and other diseases [16,17]. The global structure of this ankyrin-repeat protein is mainly stabilized by intra- and inter-repeat hydrophobic and hydrogen bonding interactions. Although lacking the Ala that contributes to the binding of GenX, the ankyrin-repeat protein could still, through the remaining same sequence that includes the His, potentially interact with GenX, which could compromise its structural stability when bound with GenX. The crystal structure of protein 36B is not available, and so Alphafold was used to predict part of the structure of protein 36B for further structural analysis. The docking was followed by MD simulation of the partial structure of the protein 36B, and GenX showed two main binding sites on the protein, site 1 and site 2 (Figure 4). Site 1 is close to the N-term (FAFPHY), where GenX is in close contact with the His13, which is consistent with the NMR results that show His contributing to binding with GenX (Figure 5). Site 2 is composed of hydrophobic and charged residues of Pro18, Arg26 and Arg30, in which the Arg30 is in close contact with the carboxyl group of GenX (Figure 6).

Further analysis of the NMR results, showed that peptide 1 (AFAFPHY), along with other amino acids in the peptide, mainly the N-term Ala and His, are significantly contributing to the binding of GenX, most likely via hydrophobic interaction and hydrogen bonds, respectively (Supplemental Figures S1, S2, S5 and S6). In referring to the NMR results, we observed the peak shifts of hydrogens of ε and δ sites (Figures S1 and S6, chemical shift 6.5 to 8.0 ppm) and the chemical shifts of both sites had a high similarity. The result strongly supports the claim that the H of NH from the imidazole ring interacts with the GenX by forming a hydrogen bond between the hydrogen and the carboxyl group of GenX. We are therefore confident that the GenX is interacting with the His13; it is very likely this is occurring via the hydrogen bond we described, even though the phenomena were not observed in the MD simulation, where we only observed that the caboxyl group of GenX is in close proximity to the NH of the imidazole ring of His13 (about 4 Å). Pro showed some shifts that were not as obvious as Ala and His (Supplemental Figures S7 and S9). Interestingly, the conformation of N-terminus of the 36B protein (MERLCSDGFAFPHY) that contains the peptide #1 showed less stability from the Alpha fold prediction when compared to the main body of the protein, and especially the very N-term end that contains the sequence of MERLC, indicating a high flexibility of the N-terminus of the 36B protein. This indicates that it cannot be ruled out that the N-terminus of 36B might be flexible enough to engage GenX in yet another (linear) conformation. Additionally, following the simulation it was observed that the very N-terminal part of MERLC showed various conformations, which was not a surprising observation. Therefore, we suspect the N-terminus of 36B remains flexible naturally, and the binding to GenX could potentially stabilize the conformation of N-terminus of 36B. After all, in the NMR measurements, the peptide was able to bind to GenX on its own, without the further context of a folded protein. In peptides #2 (APNARLS) and #4 (YTMKPNS), no strong binding was detected by 1D NMR. For #5 peptide (KSVWATT), and other amino acids in the peptides, Lys is significantly contributing to binding GenX, most likely via hydrophobic and ionic interactions (Supplemental Figures S3, S4 and S8).

Overall, this paper screened short peptides that bind with a toxic chemical GenX via phage display, and applied an MD simulation to undertake structural analysis of the protein-GenX binding positions. This paper developed a platform to screen peptides via a non-biased phage display method, which could be used in the future to screen binding partners for other toxic chemicals. For example, human proteins that contain similar sequences to the peptides identified in the paper provide information that contributes to our knowledge of how to determine the potential toxicity of GenX for human health, which strengthens calls to reduce chemical pollution in our environment. Furthermore, the identified and optimized peptides that were screened can be immobilized onto microchips that bind to and recycle GenX waste from the environment.

Overall, this paper screened short peptides that bind with a toxic chemical (GenX), via phage display, and undertook an MD simulation that applied structural analysis of the protein-GenX binding positions. This work developed a platform to screen peptides via a non-biased phage display method that could be further used to screen binding partners for other toxic chemicals. For example, human proteins that contain similar sequences to the peptides identified in the paper provide information that contributes to our knowledge of how to determine the potential toxicity of GenX to human health, which in turn assists calls to reduce chemical pollution in our environment. Furthermore, the identified and optimized peptides screened can be immobilized onto microchips that bind to and recycle GenX waste from the environment.

## 3. Materials and Methods

### 3.1. Phage Display

A commercially available PEGA resin (Acrylamide-PEG co-polymer, which contains dimethyl acrylamide and mono-2-acrylamidoprop-1-yl[2-aminoprop-1-yl] polyethylene glycol cross-linked with bis 2-acrylamidoprop-1-yl polyethyleneglycol), was used with amine as its functional group (Sigma-Aldrich, Burlington, MA, USA), and conjugated GenX to PEGA beads via a coupling reaction to form a peptide bond linkage by the carboxyl group of GenX and amine group of the PEGA bead. The reaction was then initiated by adding the activator dicyclohexyl carbodiimide (DCC) to the solution in dimethylformamide (DMF). More specifically, 200 µL of 200 mM GenX in DMSO was added to 1 mL of DMF. Then 0.06 g of activator DCC (5 folds of GenX) was added to the GenX solution. The mixed solution was then added to 1 mL of 0.4 g/mL PEGA beads in DMF (5 folds of GenX), which was then incubated at room temperature for 1 h before being dried and redissolved into 2 mL of Tris HCl pH 8.0, 150 mM NaCl, producing GenX-PEGA for the next step of phage display screening. The PEGA control beads were prepared without adding GenX.

Ph.D.™-7 Phage Display Peptide Library Kits from New England Biolabs (NEB, Ipswich, MA, USA) were used as the phage library. The phage library was diluted 100 times in Tris HCl pH 8.0, and then 500 µL of the diluted phage library was incubated with 400 µL of the previously prepared PEGA control beads for 2 h at room temperature with gentle rocking. The mixture was then spun down at 1024 RCF for 10 min at 4 °C, and the supernatant of 400 µL was then collected as the pre-screened library (phages bound to the beads were screened off in this step) for further binding with GenX. Then, 400 µL of GenX-PEGA was incubated with the 400 µL pre-screened phage library for 4 h with gentle rocking before being spun down at 1024 RCF for 10 min at 4 °C. The GenX-PEGA resin bound with phages was collected and washed 10 times, each time with 600 µL of Tris buffer (50 mM Tris pH 8.0, 150 mM NaCl, 0.1% to 0.3% Tween20) and, after each wash, spun with 1024 RCF for 1 min at 4 °C. The bound phages were eluted by incubating with 100 µL of elution buffer (50 mM Tris pH8.0, 150 mM NaCl, 1% SDS) for 10 min, before being spun down at 1024 RCF for 1 min at 4 °C. The supernatant was collected, labeled with round 1, and then amplified by following the amplification protocol from NEB, and was then ready for amplification for round 2. A total of 3 rounds were completed for the phage display panning. Titering was completed for the round 3 amplified product by following the protocol from NEB. A total of ten blue plaques (plaque 1–10) were picked for sequencing and plaques 1, 2, 4 and 5 gave us peptide sequences, and the rest were insertless clones present in Ph.D. libraries from the start. Primers of 96 sequencing gIII-96 primers were then used to sequence the inserted library sequence. The DNA sequences were translated to the peptide sequences. The peptides were ordered from a peptide making company Biomatik (Cambridge, Ontario, Canada), which completed peptide quality control with mass spectrometry and HPLC.

### 3.2. Sample Preparation and 1H NMR Analysis

The initial peptide samples, 1 mg/mL in 1 mL D_2_O, and GenX, were dissolved into a 0.1 M phosphate buffer (pH = 7.4) in D_2_O with 0.5 mM trimethylsilylpropanoic acid (TSP) to be 2 µg/mL. The initial 650 µL of peptide were transferred to 5 mm NMR tubes after being centrifuged for further NMR acquisition. The ligand titration was carried out in the NMR tube with a portion of 80 µL of GenX each time after the data acquisition. The volume was added up every time to re-calculate the concentration of the ligand in the NMR tube.

A Bruker Ascend 400 MHz high-resolution NMR with a sampleXpress autosampler was applied in this study and all the experiments were carried out using ICON-NMR software (Bruker Biospin 4.1.1), and were controlled by ICON-NMR. A 1D ZGPR experiment with water suppression was carried out with 32k increments, 64 transients. All the spectra were carefully phased and calibrated to TSP in Bruker Topspin (Bruker Biospin 4.1.1).

### 3.3. NMR Data Interpretation

All the NMR processing was carried out in Amix 4.0 (Bruker BioSpin 4.1.1) and the NMR peak lists were calculated using the peak picking function. The picked chemical shift data were exported to Excel (Microsoft) for further data analysis. The amino acid identification was based on the peak splitting patterns and the amino acids standard peaks from the Human Metabolome Database (HMDB) database and the 2D TOCSY NMR data. The ligand concentrations were corrected using the total volume before the creation of the ligand concentration and chemical shift plot.

### 3.4. Docking of the Ankyrin-Repeat-Domain-Containing Protein 36B with GenX

Since the crystal structure of human protein ankyrin-repeat-domain-containing protein 36B, which contains part of the sequence of peptide 1 is not available, Alphafold [18] was used to predict the fragment structure of 36B which includes the FAFPHY sequence. Autodock 4.2.6 and Autodock Tools [19] were used to screen GenX binding sites of 36B. More specifically, the pdbqt format files of both the GenX and protein 36B were prepared with Autodock 4. A grid centering on the protein 36B molecule was made, and the size of the grid box was set to be 120, 120, 120, to cover the whole protein molecule. The ligand was placed at a random position within the grid. Then, Autogrid was performed first to create the gpf files before the Autodock. Docking parameters were set as generic algorithm and the output was set as Lamarckian genetic algorithm (LGA) with 50 runs. Random number generator and seeds were used as docking parameters. Finally, the Autodock was performed and ligand with various poses, with estimated binding energy ranking with pdbqt file, was produced. The top ranked poses were used for further MD simulation analysis.

### 3.5. MD Simulation of Ankyrin-Repeat-Domain-Containing Protein 36B-GenX Binding Interaction

Molecular mechanics potential energy minimization and MD simulations were performed using GROMACS v.4.5.5 with the top-ranked poses from Autodock. A CHARMM36 all-atom force field was used for the simulation, and the system was solvated with an explicit water box-shaped dodecahedron in periodic boundary conditions. The system was then neutralized using the genion tool by adding sodium or chlorine ions, depending on the charge status of the molecule, followed by energy minimization, equilibration to room temperature, and MD production. More specifically, the equilibration was carried out before simulation to reduce unrestrained dynamics, where two steps of equilibration, NVT (isothermal-isochoric/canonical ensemble) and NPT (isothermal-isobaric ensemble) were performed. After the system was well-equilibrated, the production run was then performed at constant pressure (1 bar) and temperature (300 K) for 100 ns for the GenX-36B complex. The trajectory files were analyzed by using VMD [20]. The CHARMM General Force Field (CGenFF) program was used to prepare the simulation parameters of GenX [21].

## Figures and Tables

**Table 1:** The peptide sequences for the selected peptides and the measured Kd.

Peptide #	Sequence	Kd (µM)
1	**A**FAFP**H**Y	1.6
2	APNARLS	ND
4	YTMKPNS	ND
5	**K**SVWATT	1

amino acids that significantly contribute to the binding of GenX are indicated in bold.

## Data Availability

The data presented in this study are available on request from the corresponding author.

## Supplementary Materials

The following supporting information can be downloaded at: https://www.mdpi.com/article/10.3390/ijms25052686/s1.
