# Supplementary material for: Screening Peptide-Binding Partners for GenX via Phage Display
Source: Int J Mol Sci. 2024 Feb 26;25(5):2686. doi: 10.3390/ijms25052686 (PMC10932452; doi:10.3390/ijms25052686)
Supplement: Supplementary file 1 [file ijms-25-02686-s001.zip › ijms-2823307-supplementary.pdf]

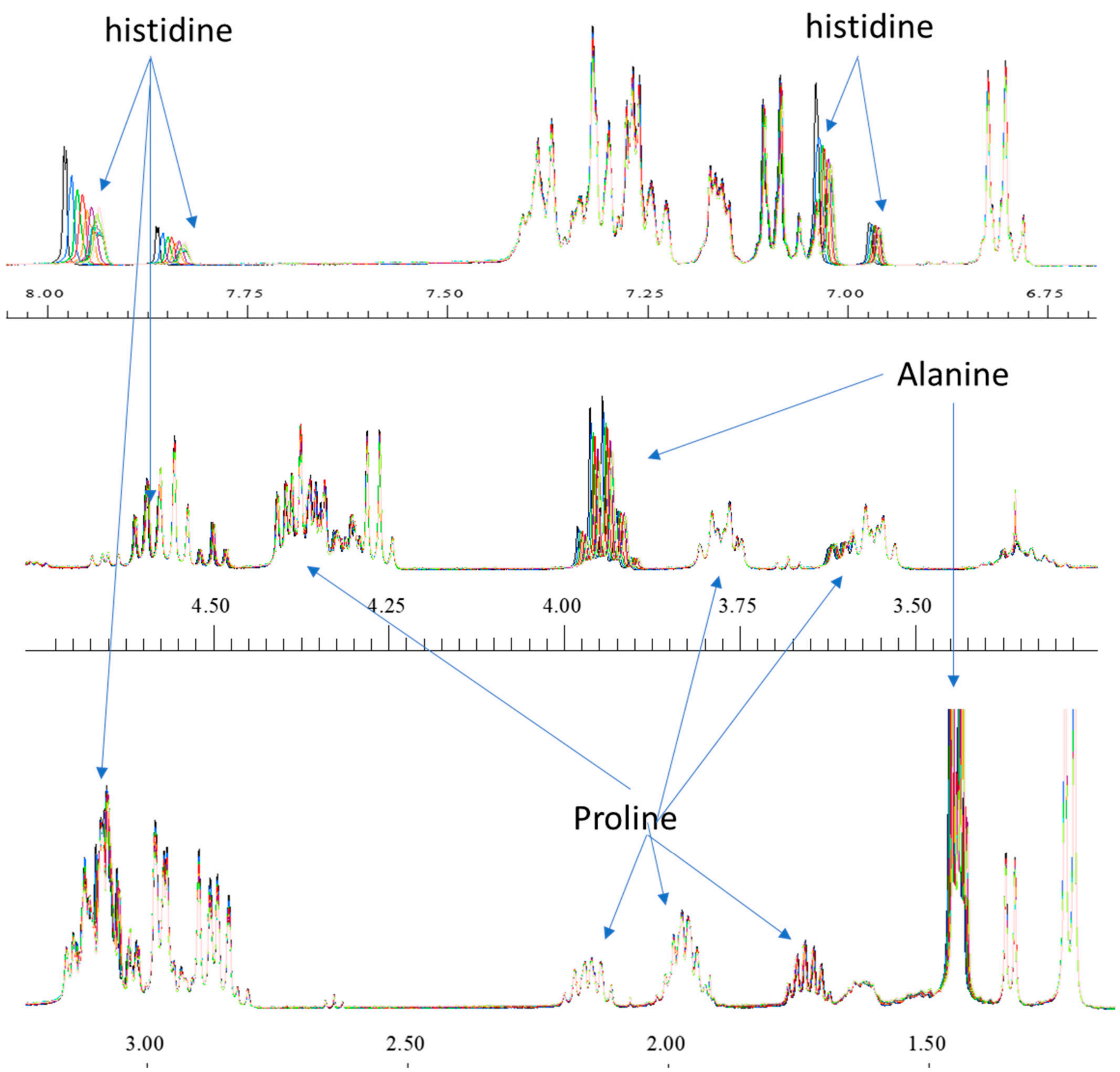

Supplemental Figure S1. The NMR overlays raw data for Peptide #1 with different concentrations of ligand titrations. One Alanine and Histidine peaks were significantly changed after titration, and proline peaks were slightly affected. The amino acids peaks were also confirmed by the 2D NMR spectrum. Alanine that has significant chemical shift changes is the N-terminal alanine judging by the chemical shift, and the position 3 alanine chemical shift was influenced by the neighbor phenylalanine (alpha carbon deshielding from 3.9 ppm to 4.3 ppm). The chemical structure and NMR peaks assignments are listed in Figures S5-S7.

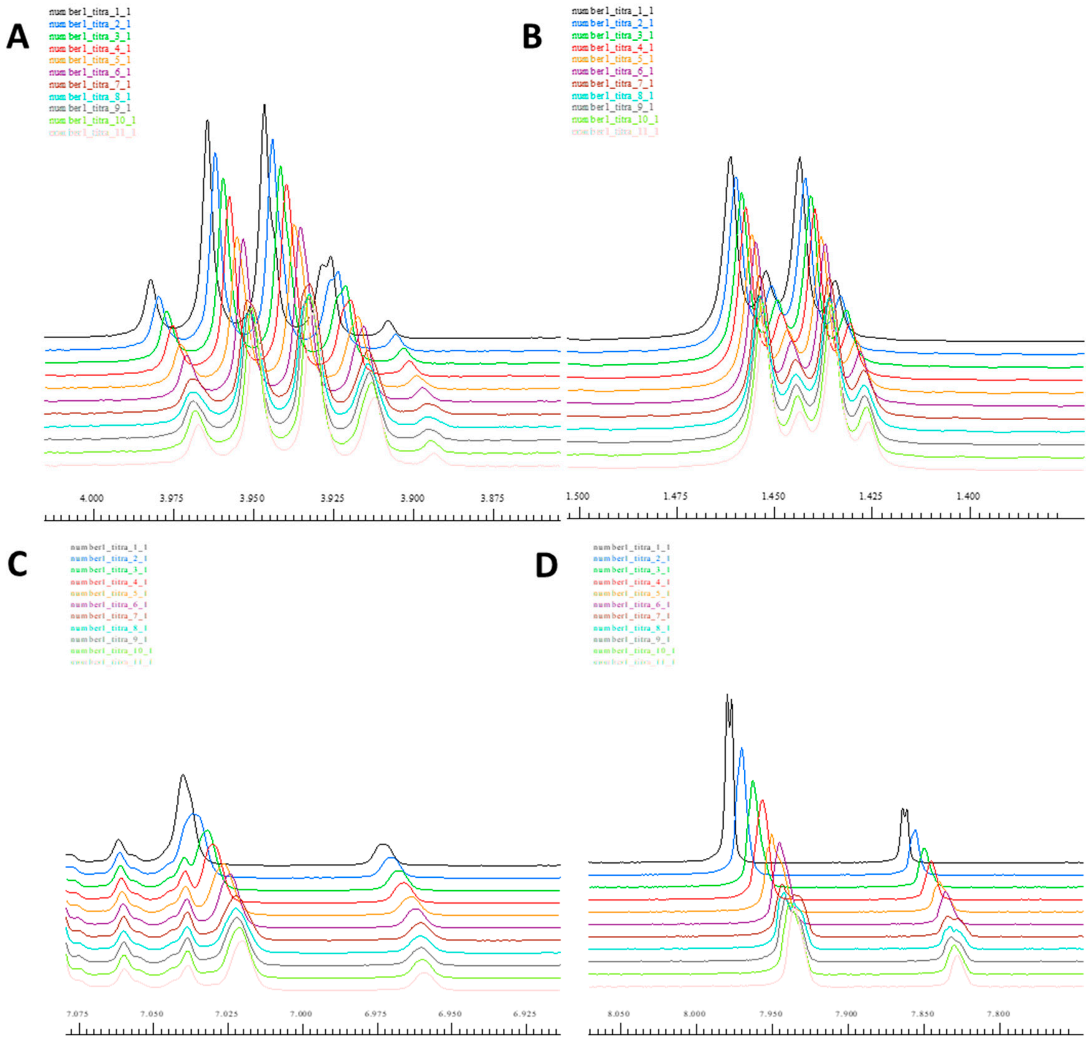

Supplemental Figure S2. The details of the Peptide #1 NMR peak chemical shift after ligand titration, and peaks were plotted from top to bottom with the increasing concentrations of ligand. A and B are peaks of N-term Alanine, C and D are peaks of Histidine.

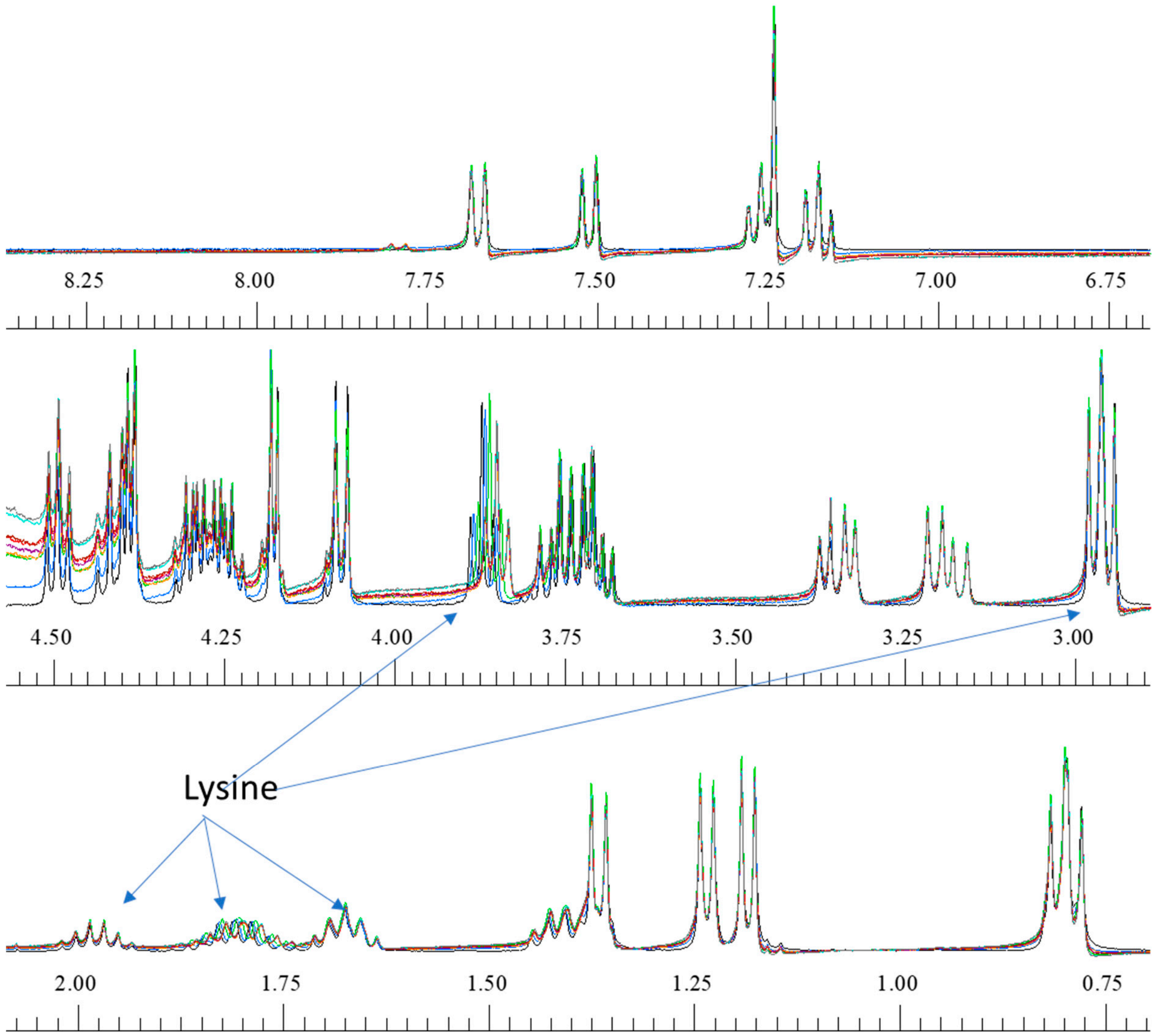

Supplemental Figure S3. The NMR overlays raw data for Peptide #5 with different concentrations of ligand titrations. Only Lysine peaks have significant NMR peak chemical shift changes. The lysine peaks were also confirmed by the 2D NMR spectrum. The lysine peak assignments are listed in Figure S8.

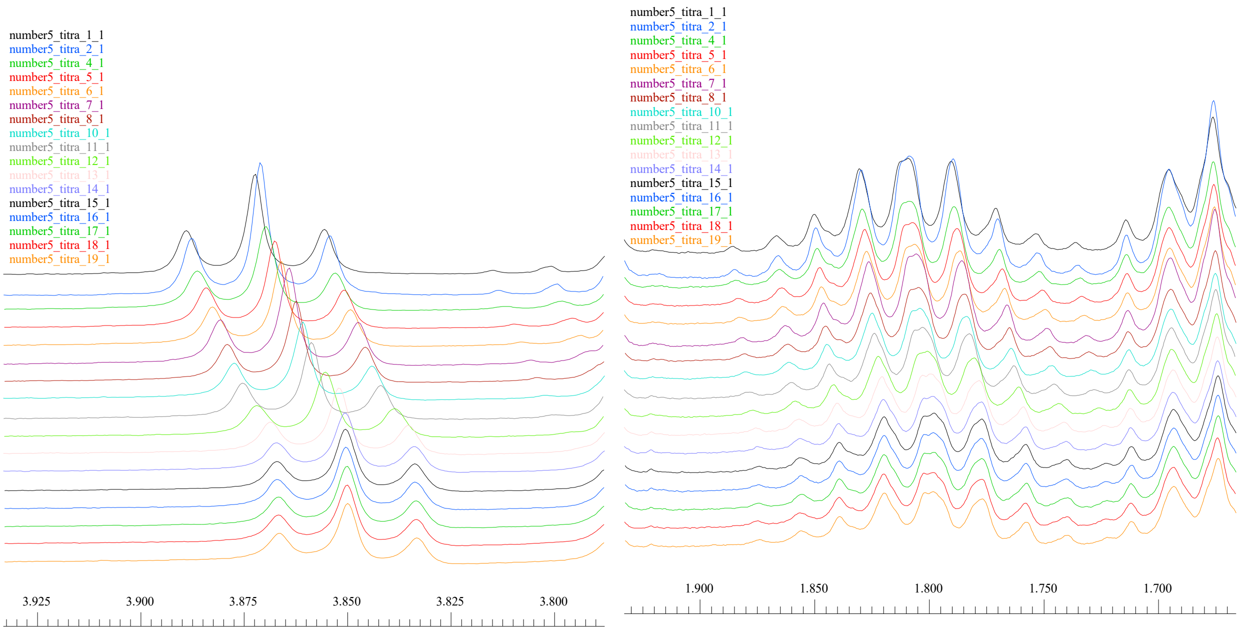

Supplemental Figure S4. The details of the Peptide #5 NMR peak (Lysine) chemical shift after ligand titration, and peaks were plotted from top to bottom with the increasing concentrations of ligand

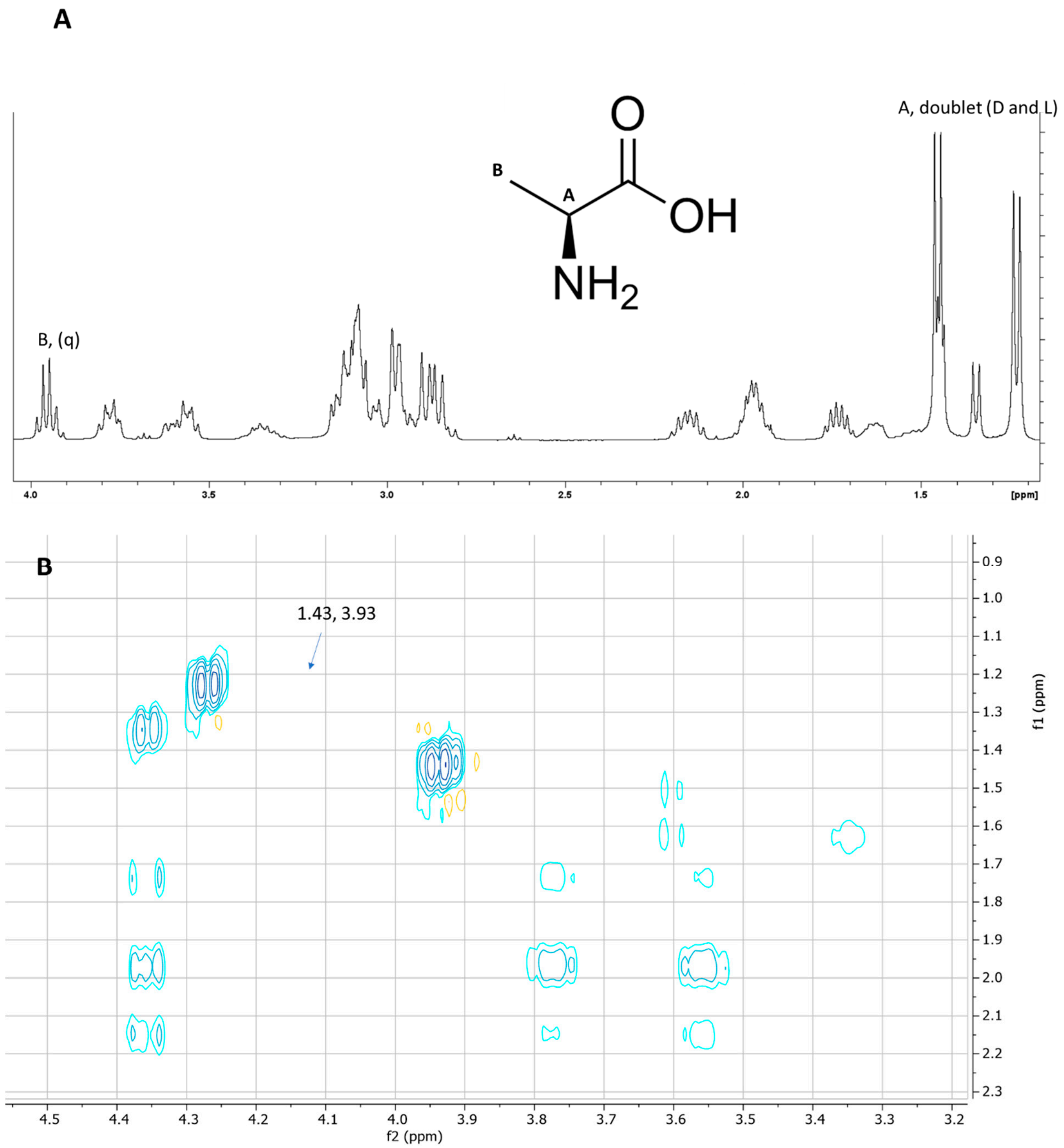

Supplemental Figure S5. A, The Peptide #1 alanine NMR spectra in Figure S1 with the assignments. B. The 2D NMR (TOCSY) spectrum of Peptide #1 with Alanine (1.4 3.9 ppm),

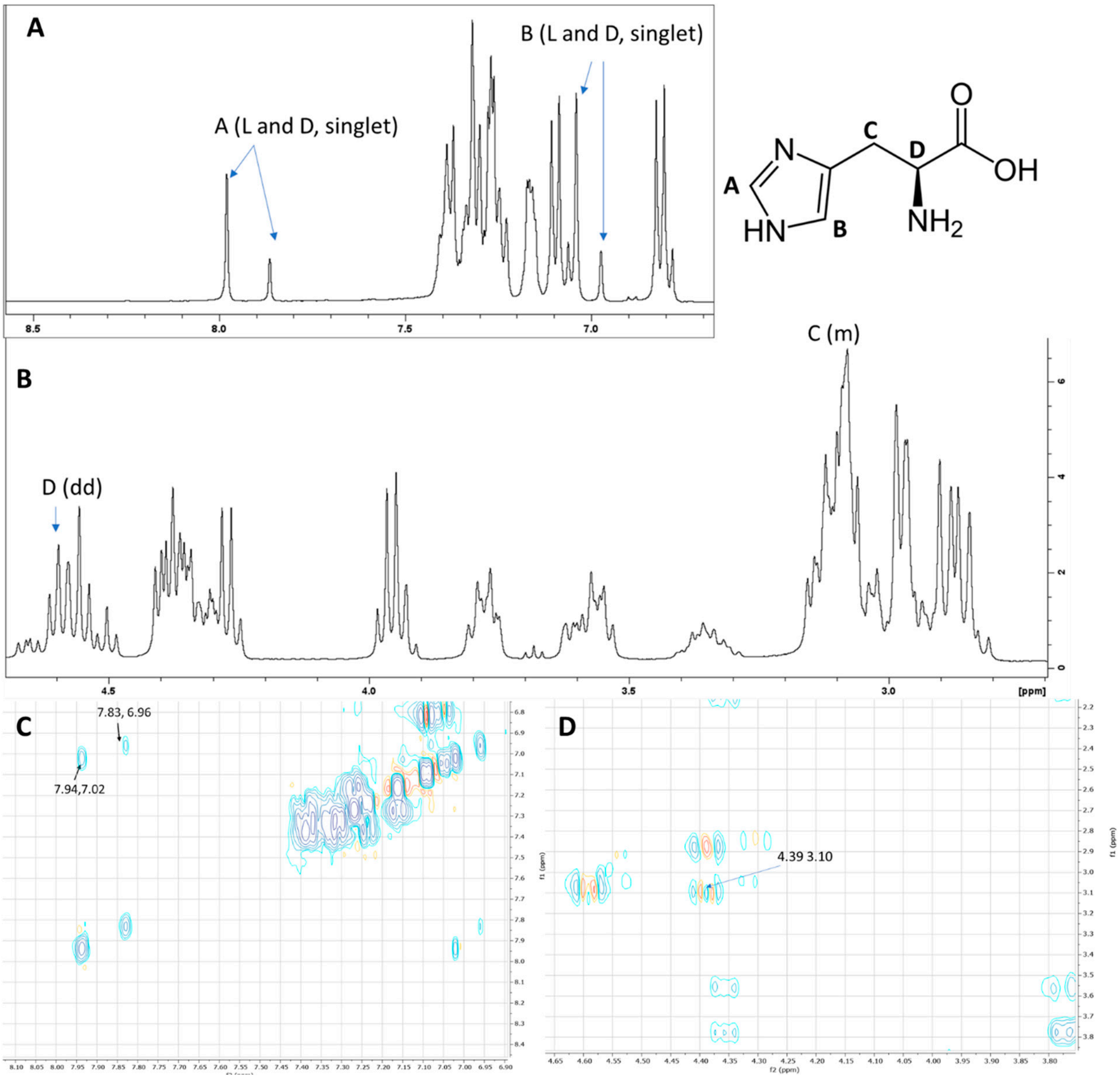

Supplemental Figure S6. A, The Peptide #1 Histidine NMR spectra with the assignments of the ones with chemical shift in Figure S1, and B the other 1D NMR peaks of the histidine assignment. C and D are the 2D NMR (TOCSY) spectrum of Histidine

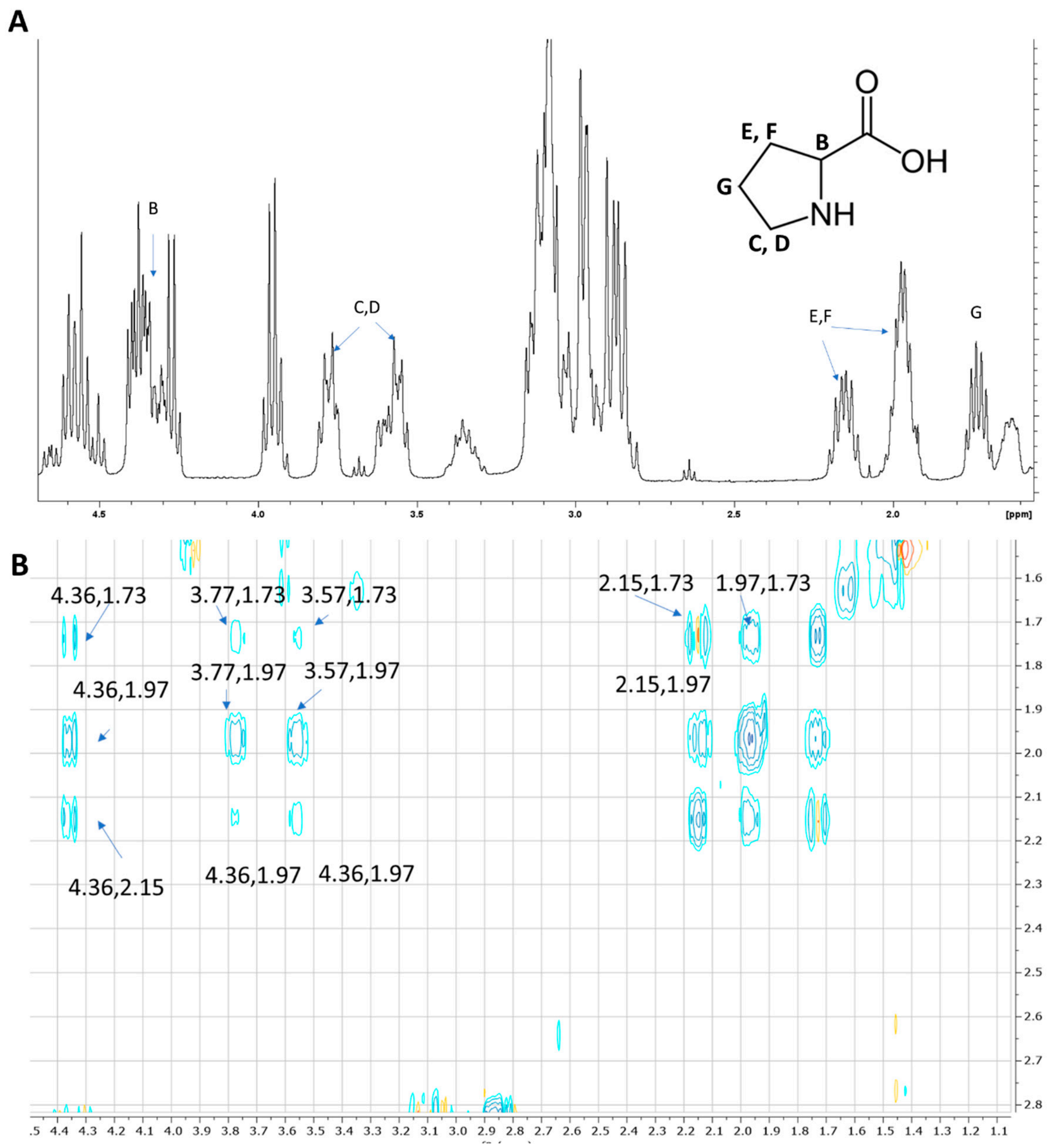

Supplemental Figure S7. A, The Peptide #1 Proline NMR spectra in Figure S1 with the assignments, and B is the 2D NMR (TOCSY) spectrum of Proline.

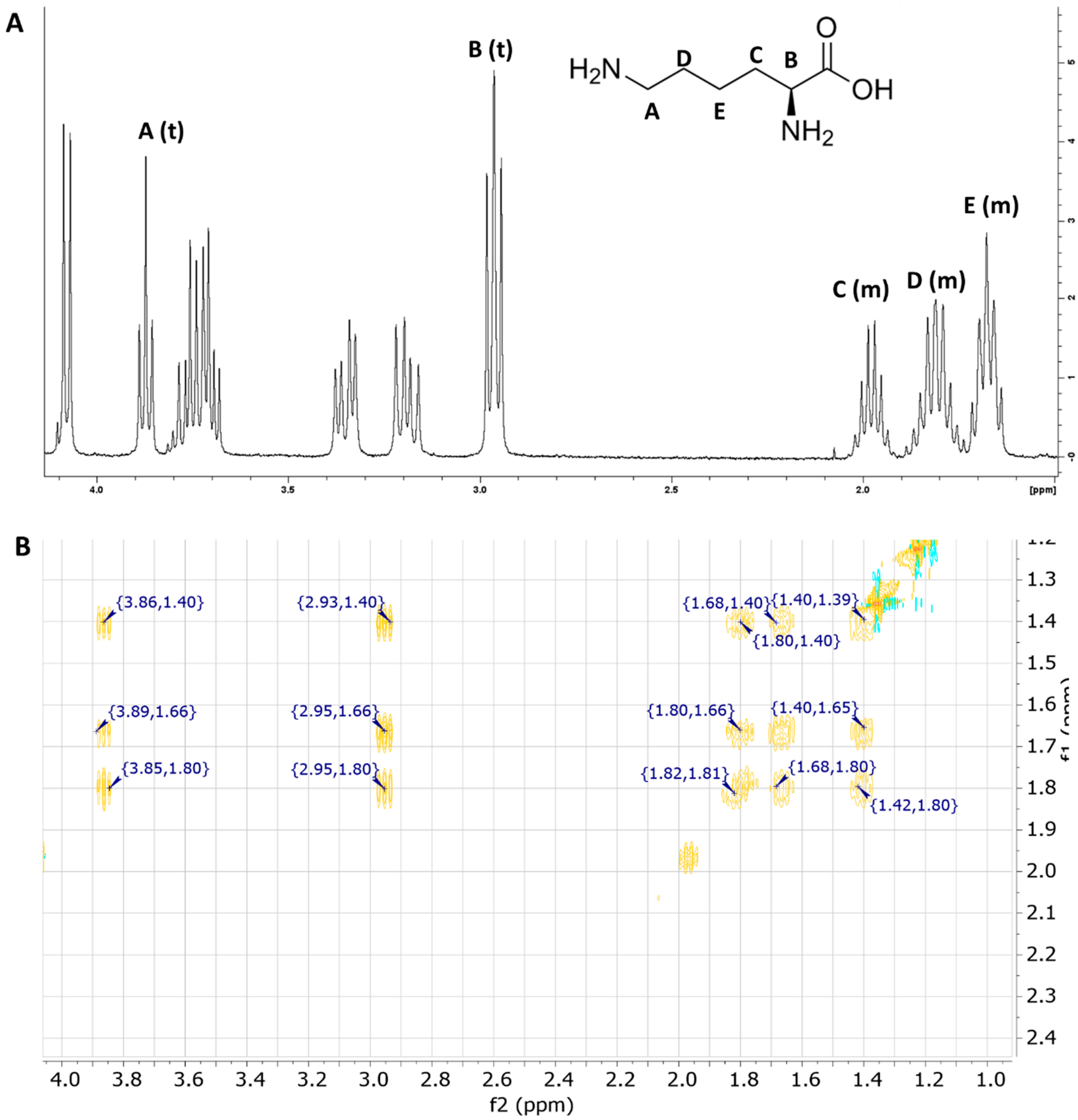

Supplemental Figure S8. A, The Peptide #5 Lysine 1D NMR spectra in Figure S3-4 with the assignments, and B is the 2D NMR (TOCSY) spectrum of Lysine.

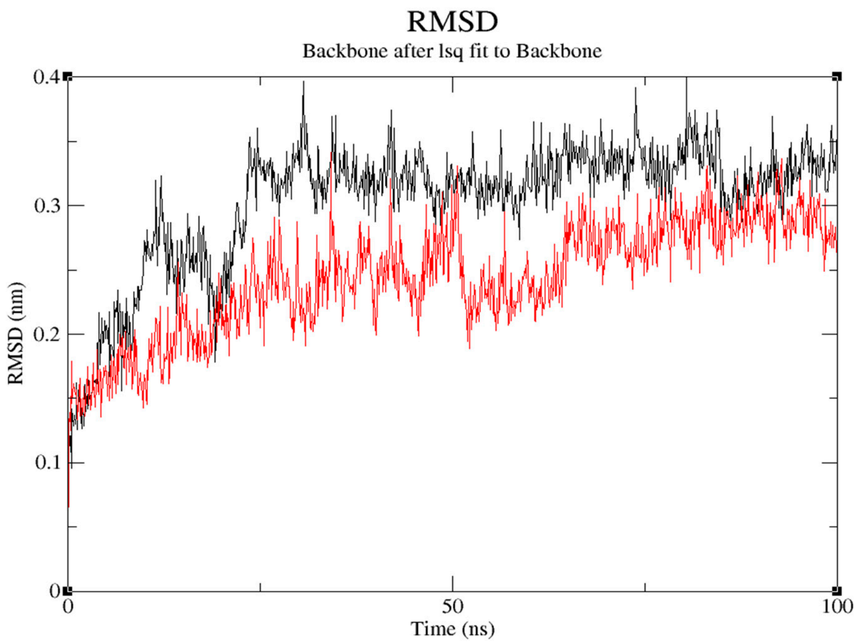

Supplemental Figure S9. The backbone RMSD of 36B bound with GenX.
